# Identification of a Novel Non-Canonical Splice-Site Variant in *ABCD1*

**DOI:** 10.3390/jcm12020473

**Published:** 2023-01-06

**Authors:** Feixia Zheng, Zhongdong Lin, Ying Hu, Xulai Shi, Qianlei Zhao, Zhenlang Lin

**Affiliations:** 1 Department of Pediatrics, The Second Affiliated Hospital and Yuying Children’s Hospital of Wenzhou Medical University, Wenzhou 325027, China; 2 Wenzhou Key Laboratory of Perinatal Medicine, The Second Affiliated Hospital and Yuying Children’s Hospital of Wenzhou Medical University, Wenzhou 325027, China

**Keywords:** adrenoleukodystrophy, protein splice variant, ATP binding cassette transporter, subfamily D, member 1, RNA sequencing, whole-exome sequencing

## Abstract

Cerebral adrenoleukodystrophy (CALD) is a fatal genetic disease characterized by rapid, devastating neurological decline, with a narrow curative treatment window in the early stage. Non-canonical splice-site (NCSS) variants can easily be missed during genomic DNA analyses, and only a few of them in *ABCD1* have been explored. Here, we studied a Chinese patient with clinical features similar to those of early-stage CALD but with a negative molecular diagnosis and a sibling who had presumably died of CALD. Trio-based whole-exome sequencing (trio-WES) and RNA sequencing (RNA-Seq) revealed a novel hemizygote NCSS variant c.901-25_901-9 del in *ABCD1* intron 1, resulting in a complex splicing pattern. The in vitro minigene assay revealed that the c.901-25_901-9 del construct contained two aberrant transcripts that caused skipping of exon 2 and a small 48-bp deletion on left of the same exon. We identified a novel NCSS variant, that extends the spectrum of the known *ABCD1* variants, and demonstrated the pathogenicity of this gene variant. Our findings highlight the importance of combining RNA-Seq and WES techniques for prompt diagnosis of leukodystrophy with NCSS variants.

## 1. Introduction

Adrenoleukodystrophy (ALD) is a rare X-linked peroxisomal disorder affecting the adrenal glands and white matter of the nervous system, with an estimated incidence of 1 in 16,000 newborns in the USA. ALD is caused by *ABCD1* mutations [1]. The most severe phenotype, childhood-onset cerebral ALD (CALD), occurs in one-third of the affected male individuals and is characterized by progressive neuroinflammatory demyelination. Boys with untreated CALD usually deteriorate to a vegetative state or die within several years after clinical onset. The well-established treatments for CALD are allogeneic hematopoietic stem cell transplantation therapy and emerging gene therapy. However, they are useful only as the first signs of abnormalities are noted on magnetic resonance imaging (MRI) of the brain, before the onset of any neurological symptoms. Thus, prompt diagnosis can improve the prognosis of CALD in boys [2,3,4,5,6].

Genetic screening using next-generation sequencing (NGS) is the most successful approach with the highest diagnostic yield in cases of underlying genetic disorders, such as CALD, and provides the fastest results [7,8]. The correct interpretation of the pathogenicity of a variant *ABCD1* is especially important. The identification of an inherited gene mutation can lead to a life-changing diagnosis of ALD in members of an extended family [9,10]. Genetic testing of X-linked ALD and screening of neonates have helped identify novel variants of unknown significance (VUSs), highlighting the need for further genetic characterization. The identification of new disease-causing *ABCD1* mutations is important for understanding the pathogenicity of new variants [1]. Here, we aimed to investigate the underlying pathogenic mechanism of a potentially unknown *ABCD1* variant that caused CALD in members of a Chinese family with a negative gene panel test. We hypothesized that trio-based whole-exome sequencing (trio-WES) and RNA sequencing (RNA-Seq) in conjunction with a minigene assay would allow us to identify the disease-causing variants of *ABCD1* in this family.

## 2. Materials and Methods 

### 2.1. Editorial Policies and Ethical Considerations

The study was approved by the Research Ethics Board of the Second Affiliated Hospital of Wenzhou Medical University (2021-K-127-02) and conducted in accordance with the Declaration of Helsinki [11]. Written consent was obtained from the patient and his legal guardians, where necessary.

### 2.2. Clinical Evaluation and Genetic Diagnosis

We documented the symptoms and signs of the disease in the proband and his family members and recorded very-long-chain fatty acid (VLCFA), adrenocorticotropic hormone, and corticosteroid levels in the plasma. Furthermore, we analyzed their brain MRI scans and the results of genetic tests, and performed trio-WES of the proband’s and his parents’ samples and RNA-Seq of the proband’s samples. Peripheral blood samples were collected from the proband and his parents for trio-WES and RNA-Seq.

### 2.3. WES and Data Analysis

WES was performed using IDT’s xGen Exome Research Panel v.1.0 on the NovaSeq 6000 platform (Illumina, San Diego, CA, USA) using paired-end reads. The reads were mapped to the GRCh38/hg38 reference genomes using a Burrows–Wheeler Aligner [12]. SAMtools [13], Picard tools (http://broadinstitute.github.io/picard/, accessed on 1 March 2020), and GATK [14] were used to call single-nucleotide variants and indels. Criteria for screening the identified variants that were considered for further analysis were as follows: (1) variants were not outside the exonic and splicing regions and not synonymous; (2) they exhibited a minor allele frequency of ≤5% based on data available on public databases; and (3) they caused protein function loss as predicted by FATHMM [15], SIFT [16], PolyPhen-2 [17], LRT [18], or MutationTaster [19]. Each variant was evaluated by using the public databases dbSNP, OMIM, HGMD, and ClinVar. The most likely disease-causing variants were prioritized according to the American College of Medical Genetics and Genomics (ACMG) classification guidelines [20] and the clinical phenotype of the patients. Sanger sequencing was performed to confirm the identified variant.

### 2.4. RNA-Seq and Data Analysis

Total RNA was extracted from the patients’ blood using the RNeasy Mini Kit (Qiagen, Valencia, CA, USA). The RNA integrity number was assessed using a 2100 Bioanalyzer (Agilent Technologies, Palo Alto, CA, USA). RNA libraries were enriched using the NEBNext Ultra™ RNA Library Prep Kit (New England BioLabs, Ipswitch, MA, USA), and 150-bp paired-end reads were sequenced. The generated reads were demultiplexed and mapped against the GRCh38/hg38 human reference genome using STAR v.2.6.1e [21]. After alignment, HTSeq [22] was used to identify the number of reads that were mapped to the genes, which involved 39,225 genes. Genes, whose 95th percentile of fragments per kilobase of transcript per million fragments mapped (FPKM) was <1, were considered non-expressed and filtered out. Aberrant gene expression was detected using the R package OUTRIDER v.1.12.0 (Bioconductor, Buffalo, NY, USA) [23,24]. The R package FRASER v.1.7.0 (Bioconductor, Buffalo, NY, USA) was used to detect aberrant splicing events [25]. Alternative pre-mRNA splicing events were analyzed using MaxEntropy (MaxEnt) software [26] and the human splicing finder (HSF) tool [27], and visualized using the Integrative Genome Viewer [28].

### 2.5. In Vitro Analysis of Splice-Site Mutations of ABCD1 Exon 2 Using the Minigene Assay

To determine whether the proband variant NM_000033.4: c.901-25_901-9 del disrupted the splicing of *ABCD1*, the sequences of intron 1 (285 bp), exon 2 (181 bp), and intron 2 (174 bp) were amplified and expressed as minigenes (exon A–intron A–multiple cloning site–intron B–exon B) in HEK293 and MCF-7 cells. Nested polymerase chain reactions (PCRs) (Table 1) were performed using genomic DNA as the initial template in order to obtain a splicing reporter of human *ABCD1* with the wild-type (wt) and the proband’s variant. The product of the first PCR was used for the second PCR. The product of the second PCR was divided into three parts. Thereafter, the third set of PCRs was performed: (1) with the primers for the restriction enzymes KpnI and EcoRI to generate the fragment of the wt; (2) using the primers for KpnI and the proband’s variant to generate the fragment MUT1; and (3) using the primers for EcoRI and the proband’s variant to generate the fragment MUT2. Next, we mixed fragments MUT1 and MUT2 (1:1) as the template for the fourth PCR and used the primers of both restriction enzymes to generate the fragment of the proband’s variant.

The fragments of the wt and the variant of the proband (mut) were inserted into the eukaryotic expression vector pcMINI to construct recombinant plasmids (pcMINI-*ABCD1*-wt/mut). They were digested and verified using gene sequencing. HEK293 and MCF-7 cells were transfected with each recombinant vector using transfection reagent Lipofectamine 2000 (Invitrogen, Carlsbad, CA, USA) and incubated for 36 h. Total RNA was extracted using the RNAiso Plus kit (TaKaRa Bio, Kusatsu, Japan) and reverse-transcribed with the Hifair™ First-Strand cDNA Synthesis SuperMix for qPCR (gDNA digester plus) (Yeasen Biotechnology, Shanghai, China). The PCR products were identified using 2% agarose gel electrophoresis and verified through sequencing.

## 3. Results

### 3.1. Clinical Description

**Figure 1:**
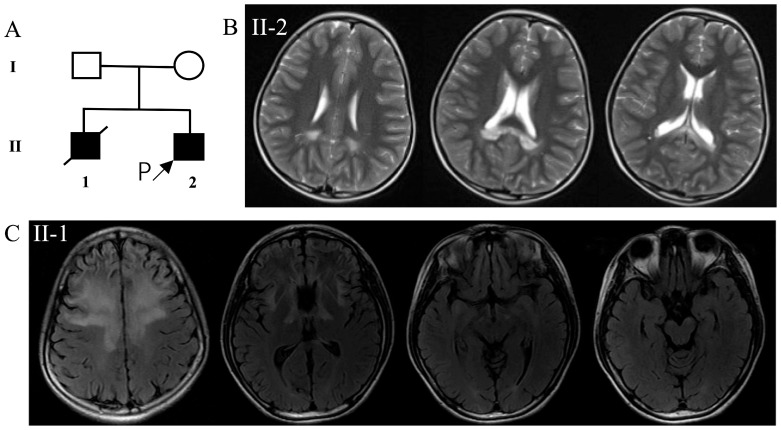
(**A**) Pedigree of the studied family and (**B**,**C**) MRI of (**B**) the proband and (**C**) his deceased brother. (**B**) T2-weighted imaging shows a high signal intensity in the splenium of the corpus callosum. (**C**) T2-weighted fluid-attenuated inversion recovery magnetic resonance imaging showing bilateral symmetrical, high signal intensity in the frontal white matter, genu of the corpus callosum, internal capsule, and mesencephalic and pontine corticospinal tracts. MRI: magnetic resonance imaging.

Herein, we describe members of a non-consanguineous family in China who presented with CALD (Figure 1A). The proband was a six-year-old boy who presented with a six-month history of headaches. The neurological examination results were normal. The MRI scans of the brain revealed mildly enlarged demyelinating lesions in the splenium of the corpus callosum (Figure 1B). He was suspected to have a parieto-occipital form of CALD. The hexadecanoic acid (C26:0) level in the plasma was 2.39 μM (normally < 1.30 μM), and the hexadecanoic to docosane acid ratio (C26:0/C22:0) was 0.040 (normally < 0.023). The adrenocorticotropic hormone and corticosteroid levels were normal. Analysis results of a leukodystrophy gene panel, containing 55 genes in total (Table S1), were negative.

The deceased elder brother of the proband showed a decline in school performance at nine years of age, followed by progressive deterioration in behavior, hearing, vision, and motor function. He died at the age of 14 years. A brain MRI (Figure 1C) performed on him at 13 years of age revealed symmetrical demyelinating lesions in the bilateral frontal white matter, genu of the corpus callosum, internal capsule, and mesencephalic and pontine corticospinal tracts. He was suspected of having had a frontal form of CALD.

### 3.2. Identification of a Novel ABCD1 Variant

Five variants, *ABCD1, CPT2, AP4B1, NDUFS1*, and *ENTPD1* (Table S2), identified using trio-WES were found to be associated with “white matter abnormalities, leukodystrophy, or very long chain fatty acid accumulation” and segregated according to the predicted genetic model (X-linked recessive, autosomal recessive, and compound heterozygote). The mutant gene *ABCD1* (RefSeq NM_000033.4: c.901-25_901-9 del) is strongly correlated with clinical measures and is a maternal variant following an X-linked recessive model of inheritance. The other four variants were considered less causative according to an autosomal recessive model of inheritance because they were present in the proband and were paternally or maternally inherited in the heterozygote form.

**Figure 2:**
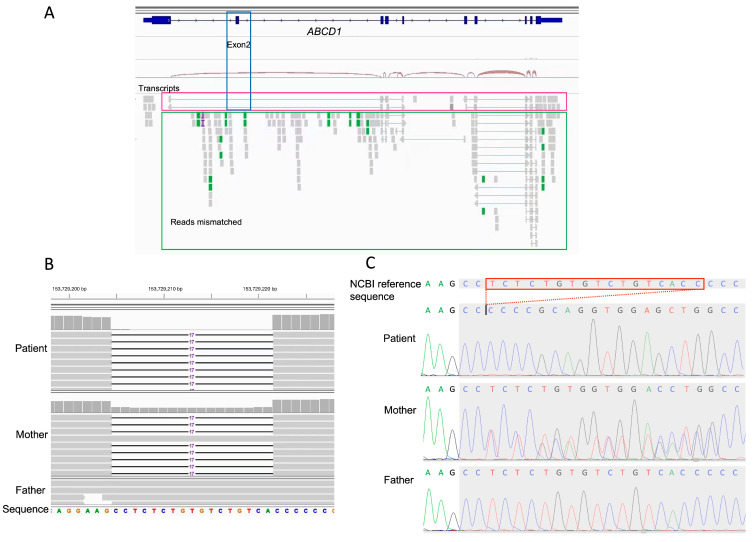
Integrated RNA- and whole-exome sequencing (seq) identified an intronic variant of uncertain significance in *ABCD1* of the proband. (**A**) RNA-Seq of the proband’s blood revealed a small number of *ABCD1* transcripts (magenta box). Exon 2 was missing among the identified transcripts (blue box) and many reads were mismatched (green box). (**B**) Trio-WES and (**C**) Sanger seq results comparing the new variant NM_000033.4: c.901-25_901-9 del of the proband with that of the heterozygous mother. No mutations were found in the father.

The RNA-Seq analysis revealed a complex splicing pattern for *ABCD1* (Figure 2A). Only a small number of *ABCD1* transcripts were identified. Examination of mRNA transcripts revealed that exon 2 of *ABCD1* was missing. NM_000033.4: c.901-25_901-9 del is likely responsible for this aberrant splicing event in *ABCD1.* HSF and MaxEnt predicted that this variation would affect mRNA splicing (−51.39% and −390.19%, respectively). This novel variant was classified as a VUS according to the ACMG PM2, PP3, and PP4 criteria [6], and confirmed using Sanger sequencing (Figure 2B). 

### 3.3. c.901-25_901-9 Del Alters ABCD1 Splicing, Causing a Loss of Transcript Heterogeneity

**Figure 3:**
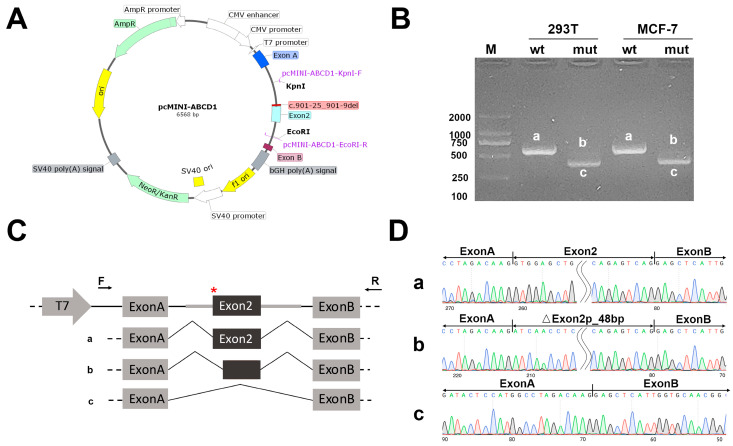
Minigene analysis inserting the wild-type (wt) and proband’s variant (mut: c.901-25_901-9 del) of *ABCD1* into the eukaryotic expression vector pcMINI. (**A**) Map of pcMINI-*ABCD1*. (**B**) Real-time PCR products of the pcMINI-*ABCD1*-wt/mut vectors in HEK293 and MCF-7 cells. Band lengths: (a) 570 bp, (b) 522 bp, and (c) 389 bp. M: DNA ladder marker. (**C**) Schematic illustration of minigene and cutting construct. (**D**) Sequencing results. Asterisk indicates exon 2 of *ABCD1*.

To test the sequencing analysis results, we performed functional in vitro experiments to study the splicing events. Different splicing patterns were observed between the c.901-25_901-9 del mutant and wt (Figure 3). The minigene of *ABCD1* wt showed a splice pattern, and an expected 570-bp-long PCR product [exon A–exon 2 (181 bp)–exon B] was identified in both HEK293 and MCF-7 cells. In contrast, the minigene of the proband’s variant (c.901-25_901-9 del) of *ABCD1* produced two aberrantly spliced products in both HEK293 and MCF-7 cells that were shorter than the wt minigene. PCR product I [exon A–exon B] was 389 bp and skipped the entire exon 2, whereas product II [exon A–△exon 2 (133 bp)–exon B] was 522 bp with a 48-bp deletion on the left of exon 2. Of the two products, product I was mainly obtained.

### 3.4. Treatment, Outcome, and Extended Family Screening

After CALD diagnosis, the proband received allogeneic hematopoietic stem cell transplantation three months after the first admission, without major adverse events. Eighteen months after treatment, the proband was going back to school. Until the last visit, 30 months after treatment, the boy had achieved smooth school performance without major functional disabilities. Cerebral demyelination was gradually arrested as determined using the brain MRI. The extended family members of the mother are healthy (Figure S1); they refused to undergo genetic testing for the identified mutation and biochemical testing for VLCFA level as they did not want the information.

## 4. Discussion

In the present study, we performed trio-WES and RNA-Seq in members of a Chinese family with two different clinical phenotypes of CALD and an uninformative multi-gene panel test. We identified the novel hemizygous VUS c.901-25_901-9 del of *ABCD1* in the proband. The proband’s mother, who carried heterozygous variants of *ABCD1*, was asymptomatic. RNA-Seq combined with the minigene assay confirmed that exon 2 skipping was the splicing pattern of *ABCD1* with the c.901-25_901-9 del variant.

In our study, leukodystrophy was considered based on family history and the sibling’s symmetrical lesions of the white matter of the central nervous system. Leukodystrophy comprises a large group of rare genetic disorders primarily affecting the white matter of the central nervous system. Disease prognosis is poor, as there is no cure in most cases. There are only a few leukodystrophies that can be cured, and this is only in the initial stages of the disease, emphasizing the need for a rapid diagnosis [8]. The process of leukodystrophy diagnosis always begins with in-depth MRI pattern recognition. Five MRI patterns have been described in patients with X-linked ALD based on the anatomic location of the initial T2 signal hyperintensity: parieto-occipital or frontal white matter, corticospinal tract, cerebellar, concomitant parieto-occipital, and frontal white matter [29]. The different MRI phenotypes of the sibling are consistent with those of CALD. Elevated VLCFA levels are indicative of peroxisomal disorder [30]. ALD is the most common peroxisomal disorder and has multiple phenotypes without an established genotype–phenotype relationship [1]. Thus, a peroxisomal disorder, specifically CALD, was first considered. 

In some cases, other peroxisomal disorders, such as Zellweger spectrum disorder [31], acyl-CoA oxidase deficiency [32], and D-bifunctional protein deficiencies [33], have been reported to present with abnormalities that show in an MRI and that resemble X-linked ALD, particularly in patients with late-onset symptoms [34]. Therefore, the leukodystrophy multi-gene panel test was the first approach in this case. As it was uninformative, a re-analysis of the possible and differential diagnoses was necessary. Although VLCFAs are regarded as the most important biomarkers for the majority of peroxisomal disorders, false-positive results have been reported in approximately 2.5% of the healthy population, hemolyzed samples, and patients with a ketogenic diet [35,36]. Therefore, other genetic disorders could not be ruled out in case the VLCFA levels were false-positive results.

As the proband was in the early stage of the disease, genetic tests needed to be optimized urgently to study the disease etiology, as the CALD therapeutic window is narrow. NGS genetic testing, including gene panel testing, WES, or whole genome sequencing (WGS), is the most successful approach for diagnosing rare diseases with the highest diagnostic yield [7,37]. In comparison to WES, WGS is more powerful for detecting variants. However, from a cost point of view, WES is more routinely used for clinical diagnostics [8,38]. Trio-WES has substantially increased the number of known leukodystrophies and improved diagnoses [7,8,39]. Nevertheless, the percentage of leukodystrophy cases without a specific diagnosis has been estimated to be 20–30% [7,39,40]. Approximately 50–75% of patients with Mendelian disorders do not receive a genetic diagnosis based on WES that could identify disease-causing variants in noncoding regions [41,42]. Most disease-causing single nucleotide substitutions in introns involve canonical +1/+2 or –1/–2 splice sites (CSSs). However, many of the identified intronic mutations are located outside the CSSs. Non-CSS (NCSS) variants are less straightforward to interpret because they exhibit considerable sequence variation and can easily be missed during genomic DNA analyses [43]. 

The majority of noncoding variants are involved in the production of aberrant transcripts through exon skipping, cryptic site use, or intron retention [44]. RNA-Seq has become an essential tool for transcriptome-wide analysis of differential mRNA splicing and differential gene expression over the past decade. Previous studies have reported that RNA-Seq helps interpret VUSs identified through WES/WGS when linked to an aberrant transcript event, and detects variants missed by current standard diagnostic approaches in rare diseases [42,45,46]. For patients in whom WES is uninformative, RNA-Seq of blood has been reported to show diagnostic utility in rare diseases by generating a 7.5% diagnostic rate and 16.7% improved candidate gene resolution [45]. WGS offers advantages over WES in the detection of such intronic or intergenic mutations, and the combination of WGS and RNA-Seq is superior in the detection and analysis of non-coding variants. WGS might be optimal for further evaluation when a patient with highly suspected genetic disorder remains undiagnosed after clinical WES [38,47]. In our study, RNA-Seq of blood samples combined with trio-WES enabled us to identify a novel NCSS variant in *ABCD1*. This variant was not detected in the leukodystrophy multi-gene panel test, which proves the importance of combining these sequencing methods in uncertain cases that might be linked to gene mutations. As of September 2022, only 43 *ABCD1* variants located in NCSSs have been reported in the *ABCD1* Variant Database (https://adrenoleukodystrophy.info/, accessed on 1 September 2022). According to the ACMG standards and guidelines, NCSS variants are considered VUSs unless their effect on splicing has been validated [20]. To date, VUSs are the most frequent NCSS variants of *ABCD1* (65.1%, 28/43). However, to the best of our knowledge, only a few mRNA studies have explored these NCSS variants in ALD [48]. In our study, the minigene assay combined with RNA-Seq confirmed that c.901-25_901-9 del causes aberrant splicing, resulting in exon 2 skipping. A likely explanation is that the variant may disrupt *ABCD1* splicing, thereby causing a premature termination of transcription and degradation of defective transcripts.

## 5. Conclusions

The combination of comprehensive DNA sequencing and RNA-Seq, and careful clinical, radiological, and biochemical evaluations, holds promise for both expediting the diagnostic process and reducing the number of unresolved leukodystrophy cases. By evaluating the pathogenic effects of the splicing variant, we gained crucial information for the assessment of a yet-to-be identified NCSS. Our findings expand the genotypic spectrum of *ABCD1* variants and provide a basis for the early diagnosis of ALD in clinical practice for the improved management of the disease to prevent negative outcomes. A limitation of the study is that we could not identify the variant and determine the VLCFA level in the extended family members of the mother. Procuring this information in the future may help confirm the pathogenicity of the variant. Another limitation is that our findings are based on a single-family study, and studies involving more cases with this variant will further validate our conclusions. 

## Figures and Tables

**Table 1:** Primers and amplification cycles used in four nested PCRs.

PCR	Primer ID	Sequence (5′–3′)	Cycle No.
**1**	2820-*ABCD1*-F	AGCACTTGGCAAACAGTGGC	30
	5038-*ABCD1*-R	CCCGCGCCCGGCTGCGTGTT	
**2**	3178-*ABCD1*-F	TGGTTTCAGTGGGAAAATGC	30
	4679-*ABCD1*-R	GCGACAGTCAGACTCCTGTT	
**3**	pcMINI-*ABCD1*-KpnI-F	GGTAGGTACCGGAGCCCAAAGAAATGGGCT	30
	pcMINI-*ABCD1*-EcoRI-R	TGCAGAATTCGAGGGGCAAGGCGAGCTCAA	
	*ABCD1*-MUT-F	CATGGCCAGGAAGCCCCCCGCAGGTGGAGC	
	*ABCD1*-MUT-R	GCTCCACCTGCGGGGGGCTTCCTGGCCATG	
**4**	pcMINI-*ABCD1*-KpnI-F	GGTAGGTACCGGAGCCCAAAGAAATGGGCT	30
	pcMINI-*ABCD1*-EcoRI-R	TGCAGAATTCGAGGGGCAAGGCGAGCTCAA	

F, forward; MUT, proband’s variant; no., number; R, reverse.

## Data Availability

The data presented in this study are available on request from the first author. The data are not publicly available due to national regulations.

## Supplementary Materials

The following supporting information can be downloaded at: https://www.mdpi.com/article/10.3390/jcm12020473/s1, Table S1. Genes in the leukodystrophy multi-gene panel. Table S2. Variants identified by trio-WES in the family. Figure S1. Pedigree of the extended family of the mother.
