# Supplementary material for: Identification of a Novel Non-Canonical Splice-Site Variant in ABCD1
Source: J Clin Med. 2023 Jan 6;12(2):473. doi: 10.3390/jcm12020473 (PMC9863105; doi:10.3390/jcm12020473)
Supplement: Supplementary file 1 [file jcm-12-00473-s001.zip › jcm-2096579-SI.pdf]

Supplementary Materials

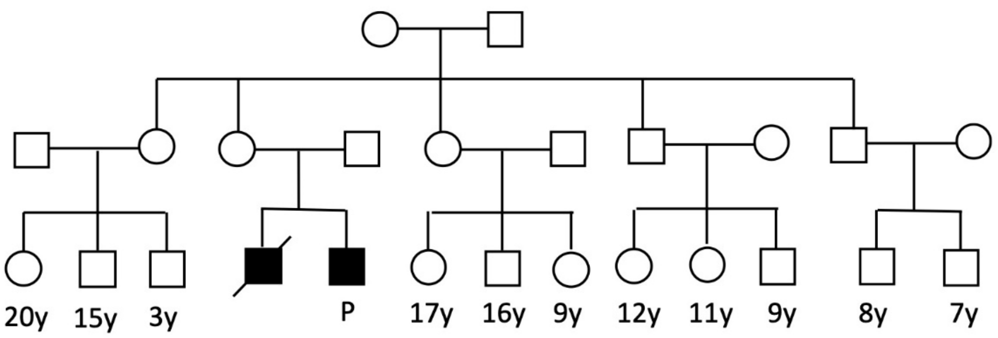

Figure S1. Pedigree of the extended family of the mother.

Table S1. Genes in the leukodystrophy multi-gene panel.

Panel	Genes
Leukodystrophy	*ABCD1, ARSA, CSF1R, EIF2B2, GJC2, PEX1, POLR3A, POLR3B, PSAP, TREX1, EIF2B1, EIF2B3, EIF2B4, EIF2B5, PLP1, GFAP, ASPA, MLC1, HEPACAM, CYP27A1, GBE1, RNASEH2A, RNASEH2B, RNASEH2C, SAMHD1, ADAR, LMNB1, SLC17A5, FUCA1, FAM126A, GALC, L2HGDH, DARS2, EARS2, SUMF1, GJA1, RNASET2, HSD17B4, ACOX1, SCP2, ALDH3A2, SOX10, MFSD8, HSPD1, MPV17, PEX10, PEX13, PEX5, PEX6, POLR1C, SDHA, SDHB, TMEM187, NOTCH3, HTRA1；*<br>*ABCD1/PLP1-MLPA*

Table S2. Variants identified by trio-WES in the family.

Gene	Variant	GnomAD MAF	ACMG classification	Model of inheritance	Zygosity		
					Proband	Father	Mother
ABCD1	NM_000033.4: intron 1: c.901-25_901-9del	--	VUS	XLR	Hemizygote	Wild type	Heterozygote
CPT2	NM_000098.3: exon 4: c.577C>T (p.Arg193Cys)	0.0018	VUS	1. AD, AR 2. AR 3. AR 4. AD, AR	Heterozygote	Heterozygote	Wild type
AP4B1	NM_006594.5: exon 10: c.1591C>T (p.Arg531Trp)	0.0013	VUS	AR	Heterozygote	Heterozygote	Wild type
NDUFS1	NM_005006.7: exon 4: c.261+9T>C	0.000004062	VUS	AR	Heterozygote	Wild type	Heterozygote
ENTPD1	NM_001776.6: exon 2: c.67T>C (p.Phe23Leu)	0.000008124	VUS	AR	Heterozygote	Wild type	Heterozygote

Trio-WES, trio-whole-exome sequencing; VUS, variant of unknown significance; XLR, X-linked recessive; AR, autosomal recessive; AD, autosomal dominant
